# Caregiver capabilities: Healthcare interventions for children with developmental disabilities

**DOI:** 10.4102/ajod.v14i0.1563

**Published:** 2025-06-26

**Authors:** Lumka Magidigidi-Mathiso, Jose Frantz, Gerard C. Filies

**Affiliations:** 1 Centre for Interdisciplinary Studies of Children, Families and Society, Faculty of Community and Health Sciences, University of the Western Cape, Cape Town, South Africa; 2 Faculty of Community and Health Sciences, University of the Western Cape, Cape Town, South Africa; 3 Interprofessional Education Unit, Faculty of Community and Health Sciences, University of the Western Cape, Cape Town, South Africa

**Keywords:** disability, developmental disability, caregivers, capacity, caregiver, interventions, healthcare, children

## Abstract

**Background:**

Developmental disabilities (DDs) involve impairments affecting children’s abilities, impacting development and necessitating specialised care. Many caregivers face challenges caring for these children, lacking access to supportive healthcare interventions. Addressing this issue aligns with United Nations (UN) goals for ensuring access to quality services for children with disabilities and their caregivers.

**Objectives:**

This study aimed to systematically review and synthesise evidence on healthcare interventions enhancing caregiver capabilities for children with DDs, identifying intervention types, components and effectiveness.

**Method:**

Our systematic review analysed peer-reviewed English-language studies from 2014 to 2024, focusing on interventions for caregivers of children with DDs. The review investigated healthcare interventions designed to enhance caregiver capabilities across diverse cultural contexts, examining international research to understand strategies supporting caregivers of children with DDs.

**Results:**

We found significant improvements in caregiver well-being through five interventions. Parent education reduces stress and improves parenting. Peer support decreased isolation while counselling enhanced family functioning. Condition-specific interventions increased intervention adherence among minorities. Combined interventions showed strong positive effects, especially when tailored. Comprehensive programmes greatly improved caregiver quality of life. Further research is needed for underserved communities and culturally adaptive interventions.

**Conclusion:**

Our review indicates potential positive parental impacts with limited evidence. Small samples warrant future research using larger studies, emphasising rigorous methods, cultural adaptation and diverse community representation.

**Contribution:**

Our review identifies promising intervention types and highlights the need for further research to optimise caregiver support and promote access to quality services.

## Introduction

### Background

Children with developmental disabilities (DDs) face immense global challenges, including in African countries with high prevalence rates (Arora et al. 2022; Olusanya et al. 2018). Developmental disabilities are a group of conditions because of impairment in physical, learning, language or behaviour areas that begin during the developmental period and typically last throughout a person’s lifetime. These include intellectual disabilities, autism spectrum disorders, cerebral palsy and other neurological disorders. Globally, it is estimated that 13.9% of children aged 2–17 years have DDs, with prevalence rates varying across regions (Olusanya et al. 2022). In low- and middle-income countries, particularly in Africa, the prevalence is thought to be higher because of factors such as malnutrition, limited access to healthcare and higher rates of birth complications. Caregivers shoulder substantial burdens, facing barriers such as limited access to services, resources and cultural stigma (Abuga et al. 2022; Akpeke, Agbemavi & Adde 2024; Nyoni 2021; Olaitan & Olaitan 2022). In our review, caregivers are defined as the primary individuals responsible for the daily care and support of children with DD. This includes biological parents, adoptive parents, grandparents and other family members who serve as primary caregivers. While we acknowledge that professionals and community members may play supporting roles, our review focuses primarily on family caregivers who bear the main responsibility for the child’s care. Cultural factors significantly influence caregiver experiences and the delivery of healthcare interventions. These include traditional beliefs about the causes of disabilities, which may attribute conditions to curses or spiritual factors, influencing help-seeking behaviours (Nyoni 2021). Additionally, community attitudes towards disability often result in social exclusion of both children and their caregivers, while family structures and gender roles can impact the distribution of caregiving responsibilities (Zuurmond et al. 2019). Healthcare interventions offer potential support through education, counselling, peer networks (Azad et al. 2022; Catalano, Holloway & Mpofu 2018), but evidence on effectiveness and cultural relevance is limited, especially in South African contexts (Constantino et al. 2022; Nyoni 2021). While some studies explore caregiver interventions in Africa (Yousafzai et al. 2022; Abuga et al. 2022), more research on context-specific, culturally relevant interventions is needed. Our systematic review aims to comprehensively explore existing healthcare interventions tailored to strengthen capabilities of caregivers of children with DD. Well-being for caregivers of children with DDs encompasses multidimensional aspects of their lives, including physical health, psychological resilience, social connectedness and economic stability (Boehm, Carter & Taylor 2021; Morin et al. 2023). It involves not only the absence of distress but also the presence of positive experiences, coping abilities and quality of life while managing the complex responsibilities of caring for a child with developmental needs (Duran, Grimsey & Arber 2022; Lunsky et al. 2021).

Interventions for parents of children with DDs strategically enhance human capabilities by leveraging multidimensional support mechanisms. These interventions focus on skill development, psychological support and empowerment, systematically transforming parental capabilities through targeted approaches (Lancaster et al. 2023; Provenzi et al. 2021). By providing contextualised training, peer support networks and practical learning strategies, interventions aim to improve parent–child interactions, reduce psychological stress and build emotional resilience (Koly et al. 2021; Szlamka et al. 2022). The most effective interventions incrementally develop caregivers’ problem-solving skills, communication strategies and self-confidence, ultimately enabling them to more effectively support their children with DDs while simultaneously enhancing their own psychological and practical capabilities (Riches et al. 2023).

Strengthening caregiver capabilities through targeted healthcare interventions has significant implications for reducing long-term social and economic burdens. When caregivers are better equipped with skills and support, there are measurable improvements in children’s developmental outcomes (Masi et al. 2021), decreased healthcare utilisation costs (Wang et al. 2022), reduced family disruption and increased caregiver workforce participation (Lavelle et al. 2023). Caregiver capability refers to the dynamic combination of knowledge, skills, psychological resources and social supports that enable caregivers to effectively meet the complex needs of children with DDs (Derguy et al. 2020; Masulani-Mwale et al. 2022). The capability approach, as applied to caregiving, recognises caregivers as agents who require certain functioning and freedoms to achieve well-being for themselves and their children (Lancaster et al. 2023; Provenzi et al. 2021). The economic impact of DDs extends beyond immediate healthcare costs to include lifetime support services, lost productivity and educational accommodations, estimated at significant costs that grow over time (Rogge & Janssen 2022). By investing in caregiver capabilities, interventions potentially mitigate these long-term societal costs while improving outcomes for both children and their families (Pickard et al. 2024).

## Methods

Our systematic review searches included PubMed, CINAHL, PsycINFO and African Journals Online (AJOL) for studies published during 2014–2024 on interventions targeting parental capabilities for caregivers of children with DD worldwide. Predefined inclusion and exclusion criteria focused the review. Data were extracted from included articles using a structured form capturing study details, interventions, outcomes, and findings. Our systematic review methodology followed the Preferred Reporting Items for Systematic Reviews and Meta-Analyses (PRISMA) guidelines (Page et al. 2021). This approach was chosen for its rigour and transparency in synthesising existing research, which is particularly suitable for evaluating healthcare interventions across diverse contexts.

The methodological quality of the included studies was assessed using Covidence. Two independent reviewers evaluated each study across key domains. Disagreements were resolved through discussion and consultation with a third reviewer. The quality assessment results were used to contextualise the strength of evidence for each intervention and to identify potential sources of bias that could impact the interpretation of findings.

**Review Question:** *What healthcare interventions are effective in enhancing the capabilities of caregivers of children with DDs, and what are their key components and outcomes?*

Population, Intervention, Comparison, and Outcome (PICO) Framework:

- **Population:** Primary caregivers (parents, grandparents or other family members) of children with DDs
- **Intervention:** Healthcare interventions aimed at enhancing caregiver capabilities (including educational programmes, counselling, peer support, combined approaches and condition-specific interventions)
- **Comparison:** Standard care, alternative interventions or no intervention
- **Outcomes:** Improvements in caregiver capabilities (knowledge, skills, psychological well-being, self-efficacy and quality of life)

### Search strategy

A comprehensive search of electronic databases was conducted. Search terms included combinations of keywords related to caregivers, DDs, interventions and capabilities. The full search strategy was developed in consultation with a research librarian.

The search strategy aimed to find published and unpublished studies on the topic by searching databases such as PubMed, Ebsco host, Health Source, Sage and University of the Western Cape (UWC) database search, which was accessed via the UWC Library, using relevant search terms such as ‘healthcare’, ‘intervention’, ‘disability’, ‘developmental disability’, ‘human capabilities’, ‘interdisciplinary team approach’, ‘parents’, ‘caregivers’ and ‘children’. The identified articles and legislative frameworks were then screened to select relevant studies for our review.

### Data extraction and analysis

**FIGURE 1:**
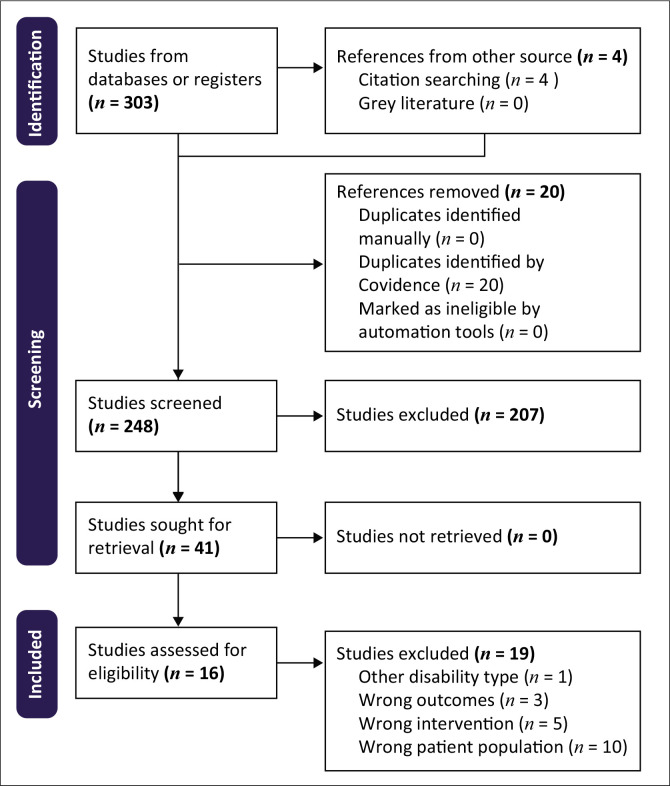
Preferred reporting items for systematic reviews and meta-analyses flow diagram.

Two independent reviewers screened titles and abstracts, followed by a full-text review of potentially eligible studies. Data extraction was conducted using a standardised form, capturing information on study characteristics, intervention details and outcomes. (Farajimakin 2024) The study selection adhered to PRISMA guidelines. From 303 initial records, 248 were screened after removing duplicates. A total of 207 records were excluded, leaving 41 for full-text review, as indicated in the flow diagram in Figure 1. Twenty-seven were further excluded based on predetermined criteria. Ultimately, 16 eligible studies were included in the systematic review, with data extracted into a structured table.

### Data analysis

We synthesised 16 studies from nine countries (2014–2024) using various designs (qualitative, reviews, mixed methods, Randomized Controlled Trials (RCTs) to examine caregiver challenges and evaluate interventions for DDs. We conducted a systematic search process, starting with database searches using relevant keywords and then screened titles and abstracts, followed by full-text reviews to ensure studies met all inclusion criteria. This rigorous process yielded 16 studies meeting all criteria and relevant to our objectives. This outcome aligns with findings from Page et al. (2021) in their guidance on systematic reviews, which notes that strict criteria often lead to a focused set of studies. Similarly, Tricco et al. (2018) in the updated PRISMA statement highlight how the systematic review process typically narrows down a large initial pool of studies to a smaller, highly relevant set. This approach ensures a comprehensive yet focused selection of literature for analysis, as emphasised by Page et al. (2021) in the updated Cochrane Handbook for Systematic Reviews of Interventions. Findings integrated caregiver experiences, effective strategies, implementation considerations, intervention effects, common themes such as feasibility and acceptability, and contextual factors influencing effectiveness and implementation.

### Eligibility criteria

Studies for the review were selected according to the following criteria.

#### Inclusion criteria

We included studies from 2016 to 2024 on interventions enhancing parenting capabilities for caregivers of children with DDs. We focused on human capabilities, interdisciplinary approaches and caregiver interventions reported in English. Qualitative, mixed methods, Systematic Review/Meta-analyses and Trial were included, with quantitative studies likely employing experimental designs such as randomised controlled trials to evaluate intervention effectiveness on outcomes such as parental stress and child development.

#### Study efficacy evaluation

Our systematic review revealed varying efficacy across interventions. Educational programmes consistently improved caregiver knowledge and skills, while psychological interventions showed immediate but not sustained stress reduction. Peer support demonstrated reliable improvements in caregiver well-being. Multicomponent, culturally adapted interventions yielded the strongest outcomes. However, small sample sizes in many studies limit generalisability, indicating the need for larger, more rigorous trials to conclusively establish intervention efficacy.

#### Exclusion criteria

The studies that were excluded were those that focused on caregivers caring for children older than 18 years of age, studies not focusing on DD and studies where adults were used as proxies for exploring youth and adolescents’ perspectives. Caregiving needs and experiences can differ significantly between caring for minors versus adults. Additionally, studies that were not focused on DD or did not include interventions were excluded, as were studies not published in English.

#### Intervention efficacy synthesis: Alignment with research question and PICO

When examining intervention efficacy through the lens of our research question and PICO framework, the synthesis reveals compelling patterns. Among the caregiver population studied, multicomponent interventions that combined educational, psychological and peer support elements demonstrated the strongest improvements in caregiver capabilities compared to single-approach interventions or standard care. Educational interventions consistently enhanced knowledge and skills (Camdena et al. 2016; Provenzi et al. 2021), while peer support interventions effectively reduced isolation and improved psychological well-being (Lancaster et al. 2023). Psychological interventions showed significant immediate stress reduction but often lacked sustained effects at follow-up (Sohmaran & Shorey 2019). The most effective interventions were those culturally adapted to local contexts, particularly in resource-limited settings where acceptability and implementation fidelity were crucial outcome determinants. Across intervention types, improvements were observed in multiple caregiver capability domains: enhanced knowledge and skills, improved psychological well-being, strengthened self-efficacy and better quality of life. However, the variability in study design, sample size and outcome measures across the included studies limits definitive conclusions about comparative efficacy, suggesting the need for more standardised, larger scale trials.

### Ethical considerations

Ethical clearance to conduct this study was obtained from the University of the Western Cape Faculty of Community and Health Sciences Biomedical Science Research Ethics Committee on 17 March 2023 (No. BM23/1/10).

## Review findings

Our systematic review synthesised 16 studies on healthcare interventions for caregivers of children with DD, published between 2014 and 2024 across nine countries. The studies utilised various methodologies including qualitative (*n* = 7), systematic reviews (*n* = 5), mixed methods (*n* = 3) and randomised controlled trials (*n* = 2) (see Figure 1). We aimed to comprehensively explore existing healthcare interventions tailored to enhance caregiver capability of caregivers with children with DDs, with a particular emphasis on African settings.

### Types of caregiver support interventions

Our review identified several types of interventions designed to support caregivers. These included parent education programmes, counselling and mental health support, peer support groups, combined approaches and condition-specific interventions. Parent education programmes focused on enhancing parental knowledge and skills (Dababnah et al. 2021), often incorporating culturally relevant content in African contexts and delivered by trained local facilitators to ensure sustainability and community ownership (Trani et al. 2020). Counselling and mental health support interventions, including individual counselling, cognitive-behavioural therapy and psychotherapy, were employed to address caregiver stress and burden (Caicedo 2016). In African settings, these interventions often integrated traditional healing practices and involved extended family members in the process (Scherer, Verhey & Kuper 2022).

Several studies highlighted the effectiveness of peer support interventions, connecting caregivers with others in similar situations (Guralnick 2019). In African communities, these groups often leveraged existing social structures and community networks. Some interventions utilised a multifaceted approach, combining education, counselling and home visits to address diverse caregiver needs simultaneously (Masulani-Mwale et al. 2019). Certain studies focused on interventions tailored to specific disabilities, such as autism, addressing the unique challenges associated with conditions (Dababnah et al. 2021).

### Analysis and synthesis of interventions

Our review analysed five key intervention approaches for enhancing caregiver capabilities. Parent educational programmes effectively built knowledge and skills through structured curricula, with greater success when incorporating practical skill-building rather than merely providing information. Counselling interventions addressed psychological impacts of caregiving, with individual counselling effective for specific psychological challenges and group counselling fostering shared problem-solving. Cultural framing significantly influenced engagement. Peer support interventions contributed uniquely through experiential knowledge-sharing and emotional validation, proving especially valuable in resource-constrained settings. Combined interventions demonstrated synergistic effects by integrating multiple support strategies, with flexible, tailored combinations showing robust improvements across caregiver capabilities. Condition-specific interventions effectively built specialised skills for particular DDs but were often less effective for broader psychosocial needs. Implementation success across all approaches depended on cultural adaptability, accessibility, trained facilitators, practical strategies, appropriate duration with follow-up, family involvement and addressing both immediate and long-term needs. The most promising interventions were contextually appropriate, addressed multiple needs and balanced immediate support with sustainable capability building.

### Duration and intensity of caregiver support interventions

The duration of interventions varied widely, ranging from short-term programmes lasting 6–8 weeks to long-term interventions spanning several months or even years. Short-term interventions typically focused on intensive parent education workshops, brief counselling interventions and time-limited peer support groups. Medium-term interventions often included ongoing parent training sessions, regular counselling appointments and facilitated peer support meetings. Long-term interventions frequently incorporated comprehensive family support programmes, ongoing mental health support, sustained peer support networks and regular home visits and follow-ups.

### Content of the intervention services to support caregivers

Content of the interventions typically covered education about specific DDs, strategies for managing challenging behaviours, techniques for promoting child development, stress management and self-care for caregivers, navigation of healthcare and education systems and building social support networks. In African settings, intervention content often emphasised integration of local cultural beliefs and practices, involvement of extended family and community members, addressing stigma and promoting community acceptance, strategies for managing resource limitations and empowering caregivers as advocates for their children.

These diverse interventions align with our study research question by demonstrating how healthcare approaches enhance caregiver capabilities across multiple domains, with the PICO elements clearly revealing that multicomponent, culturally adapted interventions produce the strongest improvements in caregiver knowledge, skills, psychological well-being and quality of life compared to standard care or single-approach interventions. These findings highlight the diverse range of interventions available to support caregivers of children with DD, with a growing emphasis on culturally adapted, holistic approaches, particularly in African contexts. The variety in duration and content reflects the complex, ongoing nature of caregiver support needs and the importance of tailoring interventions to specific cultural and community contexts.

## Implications and recommendations

Our systematic review was structured to examine the literature through the lens of the capabilities approach and interdisciplinary approach support for parents of children with DD. The primary objective was to systematically review and synthesise evidence on healthcare interventions aimed at enhancing the capability of caregivers of children with DDs, identifying intervention types, components and their effectiveness in strengthening caregiver capabilities. The diverse range of interventions identified directly addresses our research question about effective healthcare approaches for enhancing caregiver capabilities, with the PICO framework enabling systematic analysis of how these interventions impact specific caregiver populations, compare to standard care or alternatives and improve outcomes across knowledge, skills, psychological well-being and quality of life domains. From the 16 synthesised articles, we found significant improvements in caregiver well-being through five interventions mentioned below. This means that the systematic review’s findings extend beyond immediate caregivers, offering broader societal implications according to recent research. The diverse range of interventions identified directly addresses our research question about effective healthcare approaches for enhancing caregiver capabilities, with the PICO framework enabling systematic analysis of how these interventions impact specific caregiver populations, compared to standard care or alternatives and improve outcomes across knowledge, skills, psychological well-being and quality of life domains. This is substantiated by the evaluation evidence showing that educational programmes consistently improved knowledge and skills (Camdena et al. 2016; Provenzi et al. 2021), psychological interventions significantly reduced immediate stress (Sohmaran & Shorey 2019), peer support reliably enhanced well-being and reduced isolation (Lancaster et al. 2023) and multicomponent interventions that combined these approaches demonstrated the strongest and most sustainable improvements in overall caregiver capabilities (Matthews, Puplampu & Gelech 2021; Riches et al. 2023), particularly when culturally adapted to local contexts (Masulani-Mwale et al. 2019) Interventions enhancing capabilities of caregivers of children with DDs can significantly impact community healthcare infrastructure, with the PICO analysis demonstrating that effective interventions not only improve caregiver outcomes but also potentially reduce long-term social and economic burdens through decreased healthcare utilisation, improved child outcomes and increased caregiver workforce participation (Akpeke et al. 2024; Olaitan & Olaitan 2022).

### Parent educational programmes

Several studies (e.g. Studies 1, 2, 5, 9, 11, in Table 1) highlighted the importance of educational interventions enhancing parental knowledge, skills and empowerment (Dababnah et al. 2021; Masulani-Mwale et al. 2019). This PICO analysis underscores the need for government investment in training local stakeholders and community facilitators to deliver culturally competent, sustainable interventions that effectively enhance caregiver capabilities through improved knowledge, skills and psychological well-being, while promoting community ownership and addressing contextual factors that influence intervention outcomes (Trani et al. 2020).

**TABLE 1:** Characteristics of studies included in the systematic review.

Author	Title	Year	Population	Intervention type	Comparison	Outcomes	Country
Camdena et al.	Using an evidence-based online module to improve parents’ ability to manage their child with Developmental Coordination Disorder	2016	Parents of children with Developmental Coordination Disorder (*n* = 58)	Educational intervention (online module)	Preintervention baseline	Knowledge and skills improvement, behaviour change (65% reported intention to change, 50% tried strategies), improved child and family well-being	Canada
Scherr et al.	Exploring Parents’ Sensemaking Processes in the Identification of Developmental Delays and Engagement with Early Intervention Services	2020	Parents of children with developmental delays (*n* = 31)	Early intervention services	Comparison between black and white parents’ frameworks	Enhanced parent understanding of developmental delays and engagement with services	United States
Masulani-Mwale et al.	Development of a psychosocial intervention for reducing psychological distress among parents of children with intellectual disabilities in Malawi	2019	Parents of children with intellectual disabilities (*n* = 21)	‘Titukulane’ psychosocial intervention	Preintervention baseline	High acceptability and practicability of intervention when contextualised	Malawi
Reid et al.	Feasibility study of a family-focused intervention to improve outcomes for children with foetal alcohol spectrum disorder	2017	Families of children with foetal alcohol spectrum disorder (*n* = 3)	Family-focused intervention	Preintervention baseline	Improved family outcomes, positive quantitative and qualitative results	Australia
Koly et al.	Parent mediated intervention programmes for children and adolescents with neurodevelopmental disorders in South Asia	2021	Parents of children with neurodevelopmental disorders (23 studies)	Parent-mediated interventions	Various control conditions	Improved parent–child interaction, child communication, parental teaching knowledge, child developmental outcomes	United Kingdom, London
Matthews et al.	Tactics and strategies of family adaptation among parents caring for children and youth with developmental disabilities	2021	Parents of children with developmental disabilities (*n* = 39 for 46 children)	Nursing interventions	Preintervention baseline	Enhanced parental well-being, improved adaptation strategies	Canada
Davis et al.	A Capacity Building Programme to Improve the Self-Efficacy of Key Workers to Support the Well-Being of Parents of a Child with a Disability	2019	Parents and key workers (*n* = 40 parents, 13 key workers)	Capacity building programme for key workers	Stepped-wedge design	Improved key worker capacity to support parent well-being	New Zealand
Szlamka et al.	The role of advocacy and empowerment in shaping service development for families raising children with developmental disabilities	2022	Caregivers and professionals involved with children with DDs	Advocacy and empowerment interventions	Professional versus caregiver perspectives	Enhanced caregiver skills/confidence, improved service development approaches	United Kingdom
Provenzi et al.	The porridge-like framework: A multidimensional guidance to support parents of children with developmental disabilities	2021	Parents of children with developmental disabilities (*n* = 58)	Multidimensional support framework	Preintervention baseline	Significant increase in knowledge and skills, behaviour change, improved child and family well-being	Italy
Lord et al.	Determinants of parent-delivered therapy interventions in children with cerebral palsy	2018	Parents of children with cerebral palsy (17 studies)	Parent-delivered therapy interventions	Various control conditions	Identified key determinants: trusting relationships, parent coping, and prioritisation of intervention	United Kingdom
Catalano et al.	Mental health interventions for parent carers of children with autistic spectrum disorder	2018	Parent carers of children with ASD (23 studies)	Mental health interventions	Various control conditions	Improved mental health and psychological well-being, enhanced self-perspective and problem-solving	United States
Sohmaran and Shorey	Psychological interventions in reducing stress, depression, and anxiety among parents of children with developmental disabilities	2019	Parents of children with developmental disabilities (18 studies)	Psychological interventions	Various control conditions	Reduced parental stress postintervention but not at 3–6 months follow-up, inconclusive evidence for depression and anxiety reduction	Singapore
Keilty and Smith	State early intervention administrator perspectives of prenatal supports for families with high probability diagnoses	2018	Early intervention administrators (*n* = 58)	Prenatal intervention services	N/A (exploratory study)	Identified need for qualified personnel and interagency coordination	United States
Riches et al.	A study of caregiver support services: Perspectives of family caregivers of persons with intellectual disabilities in Singapore	2023	Family caregivers of persons with intellectual disabilities (*n* = 328)	Family support services	Various support models	Enhanced caregiver well-being, improved family-centred support approaches	Singapore
Massey et al.	Barriers and facilitators to parent-delivered interventions for children with or at risk of cerebral palsy	2024	Parents of children with cerebral palsy (eight electronic databases)	Parent-delivered interventions	Various implementation approaches	Identified barriers (insufficient knowledge, confidence, time) and facilitators (staff continuity, parent empowerment, flexible delivery)	United Kingdom
Lancaster et al.	Effectiveness of peer support programmes for improving well-being and quality of life in parents or carers of children with disability or chronic illness	2023	Parents or carers of children with disability (sample size: 3605)	Peer support programmes	Various control conditions	Effective for reducing stress, improving well-being and quality of life	Australia

DD, developmental disability; N/A, not applicable.

### Counselling

Mental health interventions such as counselling, cognitive-behavioural therapy and psychotherapy addressed in (Studies 6, 10, 13, in Table 1) are crucial for psychological distress, caregiver burden and coping challenges faced by parents of children with disability in African communities, indirectly enabling better childcare (Keilty & Smith 2018; Caicedo 2016). Cultural adaptation through family-centred, collective decision-making involving extended families is significant. Integrating interventions with community support systems such as groups, faith organisations or traditional healers promotes culturally relevant, holistic, sustainable and acceptable care (Scherer et al. 2022).

### Peer support

Studies 11, 12, 15 and 17, in Table 1 highlighted the value of peer support groups, mentorship and connecting parents in similar circumstances, reducing isolation and sharing strategies (Guralnick 2019). Traditional support systems such as extended families, community elders and healers can provide vital, culturally grounded support. Integrating these systems into interventions can enhance acceptability and blend traditional and contemporary approaches seamlessly, which aligns with our review research question by addressing how culturally responsive healthcare interventions can effectively enhance caregiver capabilities through improved social support structures (Masulani-Mwale et al. 2019; Nyoni 2021).

### Combined approaches

Some studies (Studies 4, 8, 14 and 16, in Table 1) examined combined interventions, combining education, counselling, coaching and home visits, targeting diverse parental needs simultaneously (Masulani-Mwale et al. 2019). Integrated care models combining healthcare, social services and community support can holistically address caregivers’ multifaceted needs. Leveraging existing infrastructures and resources enables more effective, sustainable intervention delivery (Scherer et al. 2022).

### Condition-specific interventions

Study 7 and 3 in Table 1 focused on interventions tailored to specific disabilities such as autism, addressing unique caregiver needs based on the child’s condition (Dababnah et al. 2021). These integrated community resources include culturally grounded peer support groups. The findings highlight the need for a range of supportive, tailored interventions to bolster diverse caregiver capabilities, which directly addresses our research question on effective healthcare interventions for enhancing caregiver well-being and skills (Lancaster et al. 2023; Riches et al. 2023).

### Limitations of the review

Our systematic review offers significant insights into caregiver support interventions for children with DD, especially in resource-constrained countries such as South Africa. However, it is important to recognise certain methodological constraints. The expedited nature of our review process may have limited the depth of our analysis. By prioritising studies with quantifiable outcomes and modelling approaches, such as those by Sohmaran and Shorey (2019) who conducted a meta-analysis of stress reduction outcomes, Provenzi et al. (2021) who measured specific knowledge and skill improvements, and Lancaster et al. (2023) who quantified well-being improvements in peer support programmes, we might have inadvertently overlooked other valuable research paradigms. Our reliance on a select number of academic databases potentially excluded pertinent studies from alternative sources. Furthermore, our focus on English-language publications from 2014 onwards may have overlooked earlier relevant work or non-English contributions.

Notwithstanding these limitations, our findings underscore the importance of culturally tailored interventions and pinpoint crucial areas for policy development, practical implementation and further research. These results hold particular significance for caregivers in developing nations such as South Africa, where enhancing caregiver capability is a key objective of disability response strategies. This review lays the groundwork for crafting more effective and culturally sensitive interventions for parents of children with DD. To bolster the evidence base and inform more holistic approaches, we recommend additional research that addresses the limitations. This future work will be instrumental in refining and expanding our understanding of effective caregiver support in diverse global contexts.

### Implications of the review

The findings of our systematic review have several important implications for practice and policy. Firstly, they highlight the need for culturally adaptive interventions, suggesting that policymakers should prioritise the development and implementation of programmes that are sensitive to local contexts and traditions. Quantitative evidence from Masulani-Mwale et al. (2019) demonstrated high acceptability and practicability of culturally adapted interventions, while Provenzi et al. (2021) provided measurable outcomes showing significant increases in caregiver knowledge and skills when interventions were culturally tailored. Healthcare providers and social services should be trained in culturally competent care delivery. Secondly, the varied duration of effective interventions indicates that policy should support a range of short-term to long-term support options, allowing for personalised care plans. This is supported by the quantitative findings of Sohmaran and Shorey (2019), whose meta-analysis showed stress reduction immediately postintervention but not at 3–6 months follow-up, suggesting the need for ongoing support models. Thirdly, the success of peer support groups and community-based interventions suggests that policies should facilitate and fund such initiatives, potentially through community health worker programmes. Lancaster et al. (2023) provided robust quantitative evidence through their systematic review of 3,605 participants, showing measurable improvements in caregiver well-being through peer support programmes. Finally, the integration of traditional healing practices in some settings implies that policies should consider ways to respectfully incorporate these approaches into formal healthcare systems, where appropriate. By addressing these implications, policymakers and practitioners can work towards more effective, inclusive and sustainable support systems for caregivers of children with DDs.

### Effectiveness evaluation

Our systematic review of caregiver interventions for DD revealed mixed effectiveness across approaches. Recent studies (2018–2024) showed varied outcomes in psychological interventions. While Sohmaran and Shorey (2019) found short-term stress reduction, Provenzi et al. (2021) reported better behavioural change outcomes. Successful interventions featured personalised support and multidimensional approaches. Koly et al. (2021) documented improvements in parent and child interactions, while Lancaster et al. (2023) highlighted peer support benefits. Cultural context proved crucial, with Szlamka et al. (2022) emphasising flexible intervention models. Key facilitators included staff continuity and parent empowerment. Our review indicates a need for more comprehensive, culturally sensitive support strategies.

## Conclusion

Our systematic review identified promising healthcare interventions that enhance caregiver capabilities for children with DDs (Population). The analysis revealed five effective intervention types: parent education programmes, counselling, peer support, combined approaches and condition-specific interventions (Interventions). When compared to standard care or preintervention baselines (Comparison), these interventions demonstrated measurable improvements in caregiver knowledge, skills, psychological well-being and quality of life (Outcomes). However, significant gaps remain in evaluating effectiveness across diverse cultural contexts. Future research should focus on: (1) culturally adaptive interventions for varied global settings; (2) optimal implementation strategies within existing healthcare systems; (3) targeted interventions for specific caregiver subgroups such as fathers and grandparents; and (4) condition-specific approaches for under addressed disabilities. Strengthening the evidence base requires high-quality, cost-effective studies using rigorous methodologies that measure specific caregiver capability outcomes. Interdisciplinary collaborations actively engaging caregivers in research design and implementation will be essential for developing sustainable, culturally responsive interventions that effectively enhance caregiver capabilities while accommodating resource limitations in diverse contexts. Ultimately, this approach holds promise for improving both caregiver well-being and child outcomes while potentially reducing long-term social and economic burdens.
